# Changes in the Polyphenolic Profile and Antioxidant Activity of Wheat Bread after Incorporating Quinoa Flour

**DOI:** 10.3390/antiox11010033

**Published:** 2021-12-24

**Authors:** José Vicente Gil, Adelaida Esteban-Muñoz, María Teresa Fernández-Espinar

**Affiliations:** 1 Department of Food Biotechnology, Institute of Agrochemistry and Food Technology (IATA-CSIC), 46980 Paterna, Valencia, Spain; jvgil@iata.csic.es; 2 Food Technology Area, Faculty of Pharmacy, University of Valencia, 46100 Burjassot, Valencia, Spain; 3 Departament of Nutrition and Bromatology, Campus de Cartuja, University of Granada, 18071 Granada, Granada, Spain; aidaem@ugr.es

**Keywords:** *Chenopodium quinoa*, black quinoa flour, free and bound polyphenols, UPLC-MS, phenolic compounds, bread making, antioxidant activity

## Abstract

Quinoa is a trend and a promising functional food ingredient. Following previous research into the impact of incorporating quinoa flour on the polyphenol content and antioxidant activity of bread, this study aimed to bridge an existing gap about the qualitative and quantitative polyphenolic profiles of such bread. The UPLC-MS/MS analysis showed that quinoa bread, made with 25% quinoa flour of a black variety, presented more compounds than refined-wheat bread, and levels were remarkably higher in many cases. Consequently, the quinoa bread presented clearly improved polyphenolic content than the wheat bread (12.8-fold higher considering the sum of extractable and hydrolyzable polyphenols), as supported by greater antioxidant activity (around 3-fold). The predominant compounds in the extractable fraction of quinoa bread were *p*-hydroxybenzoic acid and quercetin (50- and 64-fold higher than in wheat bread, respectively) and rutin (not detected in wheat bread), while ferulic and sinapic acids were the most abundant compounds in the hydrolyzable fraction (7.6- and 13-fold higher than in wheat bread, respectively). The bread-making impact was estimated, and a different behavior for phenolic acids and flavonoids was observed. Extractable phenolic acids were the compounds that decreased the most; only 2 of 12 compounds were enhanced (*p*-hydroxybenozoic and rosmarinic acid with increments of 64% and 435%, respectively). Flavonoids were generally less affected, and their concentrations considerably rose after the bread-making process (7 of the 13 compounds were enhanced in the extractable fraction) with especially noticeably increases in some cases; e.g., apigenin (876%), kaempferol (1304%), luteolin (580%) and quercetin (4762%). Increments in some extractable flavonoids might be explained as a consequence of the release of the corresponding hydrolyzable forms. The present study provides new information on the suitability of quinoa-containing bread as a suitable vehicle to enhance polyphenols intake and, hence, the antioxidant activity in daily diets.

## 1. Introduction

Polyphenols are well-known phytochemicals with antioxidant properties [1]. Their regular intake can alleviate or help to prevent different diseases typical of the modern era. We specifically refer to noncommunicable diseases linked with lifestyle choices [2,3]. Given government institutions’ recommendations on health issues and the scientific advances that support dietary interventions to control certain lifestyle chronic diseases, consumers have been drawn to polyphenols consumption. Food manufacturers have seen a new market opportunity in this situation, which currently focuses especially on launching new products capable of increasing dietary polyphenols intake.

Bread is a popular worldwide food characterized by its daily consumption. Thus it has the potential to easily reach a large part of the population [4]. It is also easily reformulated food. For these reasons, bread is one of the preferred foods by the industry to act as an effective vehicle for a diversity of ingredients, including those considered a source of health-promoting compounds such as polyphenols [5]. Quinoa (*Chenopodium quinoa* Willd.) is a grain-like trendy food ingredient in which growing interest has been shown in recent years. This pseudocereal is a suitable source of nutrients, fiber, and bioactive compounds with interesting antioxidant activity for healthy diets [6,7]. Quinoa has proven human health-promoting properties [6,7,8], and the possibility of incorporating it into new functional foods has been explored [9,10]. Quinoa seeds offer the peculiarity of being ground, and whole flours can be used in bakery products to completely or partially substitute refined wheat. The effect of adding quinoa flour on bread’s technological parameters and nutritional properties has been studied [10,11,12,13]. These studies show improved nutritional quality of quinoa bread (QB) made by substituting 10–25% of wheat flour (WF) compared to 100% wheat bread (WB). A decrease in technological bread quality has been described but is slight and does not negatively impact consumer acceptability. Therefore, quinoa flour (QF) has been proposed as a feasible ingredient in bakery products. The successful impact of quinoa-containing bread on polyphenol content and antioxidant activity contents has been addressed, but studies are still scarce [14,15,16,17]. To our knowledge, the studies reported to date have not taken into account the polyphenolic profile of quinoa-containing WB. Thorough knowledge of the polyphenolic composition and content of a food matrix is essential information for properly assessing their bioavailability [18]. Each polyphenol type undergoes different enzymatic and microbial changes during digestion that define the way they are absorbed and used by the body [19]. Bioavailability is one of the main key factors to consider the polyphenol-rich food consumption capacity to promote positive health effects [20]. Regarding the gut microbiota, it is increasingly evident that this is linked to polyphenol bioavailability and bioactivity, as recently reviewed [21,22].

Based on these remarks, a detailed identification and quantification of polyphenolic compounds in functional products are necessary as a first step to establishing conclusive evidence for their effectiveness. The present work delved into the effect of adding QF on the polyphenolic profile of WB and, thus, on its antioxidant content. The study focused on quinoa breads containing 25% QF of a black variety. In previous work, we showed the potential of colored varieties to improve bread’s total polyphenolic content and antioxidant activity [17]. UPLC-MS/MS was used for the identification and quantification of individual phenolic compounds in soluble-free and insoluble hydrolyzable bound fractions in raw materials (QF and WF) and in bread (quinoa and control wheat). The consideration of soluble and bound forms more realistically reflects the contribution to final polyphenol intake. The reason for this is that polyphenols in both forms could exert a health impact depending on the diverse gut events that they undergo, and therefore, none of them should be ignored [23,24]. The results provided information about differences between QB and WB and contributed new knowledge about polyphenolic content and antioxidant capacity improvement in quinoa-containing bread. In addition, the effect of the bread-making process was also examined since changes in the number and concentration of individual compounds are an important factor that influences the bioavailability and, thus, the biological activity of a food matrix upon consumption [19]. As far as we know, this is the first time that such estimation has been made on refined WB partially enriched with this ingredient.

## 2. Materials and Methods

### 2.1. Chemical, Reagents, and Quinoa Samples

Black quinoa seeds (Organic quinoa Real^©^) were supplied by Ekologikoak (Bizkaia, Spain). QF was obtained by employing a coffee grinder (Aromatic, Taurus, Oliana, Spain) and used immediately. WF and commercial baker’s dried yeast were purchased from a local market.

The reagents used to measure antioxidant capacity were: DPPH (2,2-diphenil-1-picryl hydrazyl) supplied by Sigma-Aldrich (St. Louis, MO, USA); TPTZ (2,4,6-tri(2-pyridyl)-s-triazine) for the FRAP assay and Trolox (standard curves) were supplied by Acros Organics (Geel, Belgium). The reagents for the extraction of phenolic compounds and samples preparation were acetone, methanol, and ethanolamine, and were supplied by Acros Organics. Formic acid came from Sigma-Aldrich. Sulfuric acid and acetonitrile and acetic acid, both of HPLC grade, were obtained from Panreac Química SL (Barcelona, Spain).

Commercially pure standards were acquired for quantitative purposes. Benzoic acid, *p*-hydroxybenzoic acid, 2,4-dihydroxybenzoic acid, *p*-coumaric acid, vanillic acid, gallic acid, caffeic acid, ferulic acid, syringic acid, sinapic acid, daidzein, apigenin, naringenin, luteolin, catechin, epicatechin, quercetin, and epigallocatechin were purchased from Sigma-Aldrich. Kaempferol, chlorogenic acid, rosmarinic acid, kaempferol-3-*O*-D-glucoside, quercetin-3-*O*-glucopyranoside, hyperoside, and rutin were supplied by Extrasynthese (Lyon, France)

### 2.2. Bread-Making Process

The control WB and the black QB were prepared and formulated as previously described [17]. The wheat dough formula was: 450 g of wheat flour, 2.5 g/100 g flour basis of dried yeast, 6 g/100 g flour basis of sodium chloride, and 2.5 g/100 g flour basis of distilled water. For the quinoa bread, WF (25%) was replaced with black QF (water absorption, 58.7 g/100 g flour basis). Bread types were prepared in a breadmaker (PN500, Ufesa, Spain) following the manufacturer’s instructions. The pre-established baking program 1 and the medium toasting level were applied. The obtained bread types were dried at 40 °C for 3 h by forced-air convection oven drying (Binder, Germany) and ground to a fine powder in a domestic mincer (Moulinex, Ecully, France).

### 2.3. Extractable and Hydrolyzable Polyphenols Fraction Extraction

The extractable polyphenols fraction (EPF) and the hydrolyzable polyphenols fraction (HPF) were obtained according to the work of [25] with modifications. To obtain the EPF, 0.25 g of flours (wheat and quinoa) and the ground bread were processed in two consecutive 1 h incubation steps at 24 °C with shaking at room temperature with 10 mL of acidic methanol/water (50:50, *v*/*v*; pH 2) and 10 mL of acetone/water (70:30, *v*/*v*). Samples were centrifuged at 8000× *g* after each incubation step. Both supernatants were combined, concentrated to dryness in a rotary vacuum evaporator at 35 °C, and reconstituted in 1 mL of methanol/water 50:50 *v*/*v* with 0.1% formic acid. The final extracts were filtered through a 13 mm, 0.20 µm membrane filter prior to the UPLC analysis.

The HPF was obtained by acidic hydrolysis by incubating the resulting residues with 20 mL of methanol/concentrated sulfuric acid (9:1) at 85 °C for 20 h and subsequently vacuum-filtering through Whatman No. 1 filter paper using a Büchner funnel. pH was adjusted to 5.5 with ethanolamine, and distilled water was added up to a final volume of 50 mL. Hydrolyzable polyphenols were then extracted by solid-phase extraction (SPE) with Oasis HLB Waters cartridges (6000 mg, 35 cc, 60 µm) (Milford, MA, USA) previously activated with 50 mL of methanol and 50 mL of 0.1% formic acid in water. The sample (50 mL) was then loaded, washed with 60 mL of 0.1% formic acid in water, and eluted with 60 mL of methanol and 60 mL of 80% methanol in water. The combined extracts were concentrated to dryness in a rotary vacuum evaporator at 35 °C and reconstituted in 1 mL of methanol/water 50:50 *v*/*v*. The final extracts were filtered through a 13 mm, 0.20 µm membrane filter prior to the UPLC analysis.

### 2.4. UPLC-MS/MS Analysis

Phenolic compounds were identified and quantified by a UPLC-MS/MS analysis using an Acquity UPLC HSS T3 1.8 µm column, with a gradient elution consisting of water containing 0.1% (*v/v*) acetic acid (solvent A) and acetonitrile (solvent B) for 25 min at a flow rate of 0.4 mL/min. The solvent gradient was: at 0 min 5% B, 15–15.10 min 95% B, and 15.10–25 min 5% B (re-equilibration step). Phenolic compounds were identified in a Waters SYNAPT G2 HDMS Q-TOF high-resolution spectrometer by comparing the retention times of peaks and the fragmentation data in samples to those of 25 standards (Section 2.1). Quantification was performed using a Waters ACQUITY I CLASS model chromatograph instrument (Waters, Mississauga, ON, Canada) equipped with a Waters XEVO TQ-XS. Ionization was performed by UniSpray (US). Individual compounds were quantified by constructing calibration curves with commercial standards in a concentration of 1–1000 µg/L.

### 2.5. Antioxidant Activity Assays

Antioxidant capacity was determined by two spectrophotometric assays: DPPH (α-diphenyl-β-picrylhydrazyl free radical scavenging method according to the work of [26]; FRAP (ferric-reducing antioxidant power assay) in line with [27]. The original protocols were adapted to the microplate format as previously described [17]. Determinations were made in triplicate in each extract by a microplate spectrophotometer reader (SPECTROstar Nano, BMG LabTech, Ortenberg, Germany). The results were expressed as µmol of Trolox equivalents (TE)/g sample dry (d.m.).

### 2.6. Statistical Analysis

The Student’s *t*-test (Microsoft Excel 2010) was used for the pairwise comparisons between means. Significant differences were considered at a *p*-value of at least < 0.05. Data are presented as the mean ± standard deviation of three independent repetitions.

## 3. Results and Discussion

The total polyphenolic content, estimated by a spectrophotometric assay, of the black QF and the resulting 25% QB, and their comparison to WF and the control bread (WB), were reported in a previous study [17], where we showed the potential interest of black QF as a natural antioxidant ingredient in bread making. Here the EPF and HPF extracts were characterized by UPLC-MS/MS, and their polyphenolic compounds were identified and quantified. Table 1 shows the 25 polyphenolic compounds identified in the EPF and HPF of the raw materials (WF and QF) and breads (WB and QB), 12 of them were phenolics acids (PAs) (7 hydroxybenzoic acids (HBAs) and 5 hydroxycinnamic acids (HCAs)), and 13 were flavonoids (FLs). The 25 identified compounds were present in the EPF, while only 13, 7 of which were PAs and 6 FLs, were found in the HPF.

### 3.1. Quantification of the EPF and HPF Phenolic Compounds in Raw Materials

As a basis for comparisons, raw materials were analyzed. The contents of the individual compounds identified in both extracts (EPF and HPF) are shown in Table 2 (PAs) and Table 3 (FLs). When considering the sum of the individual compound concentrations in each major polyphenol category, QF presented significantly higher contents of PAs and FLs than those quantified in wheat. It is worth noting that FLs content was around 55-fold higher in the EPF of QF. These results clearly indicate the potential of the black QF to improve the total polyphenols (PPs) content of wheat-based baking products.

Although WF and QF shared 19 compounds in the EPF, 10 PAs, and 9 FLs, most of them were by far more abundant in QF. The differences in the *p*-hydroxybenzoic acid concentration were especially noteworthy for being 26.1-fold higher in QF than in WF, as were those of *p*-coumaric (155.7-fold higher) and of flavonoids luteolin, naringenin, quercetin, and rutin (18.9-, 58.7-, 228.6-, and 92.7-fold higher in QF, respectively). In the HPF, 2-4-dihydroxybenzoic, syringic, and ferulic acids were around twice as abundant in QF than in WF. Some compounds were identified only in QF (EPF: hyperoside, kaempferol, and quercetin-3-*O*-glucopyranoside, HPF: rosmarinic acid, sinapic acid, luteolin, and quercetin), and sinapic acid stood out for its very high concentration (223 µg/g). All the compounds identified in the EPF of QF, except 2,4-dihydroxybenzoic acid, have been reported for other quinoa seeds in this fraction [6,28,29,30,31]. The occurrence of *p*-hydroxybenzoic and *p*-coumaric as the main phenolic acids of the EPF quinoa flour (76% and 19% PAs_EPF_ content, respectively) has also been described by other authors, but they detected them at lower concentrations [28,29,30,31,32]. Rutin, the principal flavonoid (around 70% of the FLs_EPF_ content), was among the main compounds in other quinoa seeds and at similar concentration levels [31,33,34]. Studies that have contemplated the polyphenolic profile of the HPF of quinoa are scarce. Of the nine compounds that were identified, five (2,4-dihydroxybenzoic, syringic, *p*-coumaric, ferulic, quercetin) have been reported in this fraction elsewhere [29,31,35,36,37]. Syringic and ferulic acids were identified among the main compounds in the HPF (17% and 60% of the PPs_HPF_ content, respectively), as previously described by other authors for certain varieties [35,36,37], but the present study detected them at higher concentrations. Discordant concentration levels to those reported in the literature are also addressed by other authors and can be explained by multiple factors [38]. As far as we know, rosmarinic acid, sinapic acid, luteolin, and naringenin are herein identified for the first time as HPF components in quinoa. Sinapic acid came at a remarkable concentration in this fraction and accounted for up to around 23% of PPs_HPF_ content.

### 3.2. Quantification of the EPF and HPF Phenolic Compounds in Bread

When bread extracts were analyzed, the number of compounds was larger in QB than in WB (control); for the EPF: 22 compounds in QB vs. 16 in the control; for the HPF: 9 vs. 6 (Table 1). QB and WB shared some compounds, specifically 16 of the 22 compounds detected in the QB EPF also appeared in the control bread, but nine of them at a higher concentration in QB (between 2.3- and 64-fold statistically significantly greater in QB than WB; Table 2 and Table 3); the largest increase corresponded to quercetin. Only chlorogenic and apigenin were more abundant in WB, and five compounds (2,4-dihydroxybenzoic, syringic, daidzein, epigallocatechin, and luteolin) appeared at not significantly different concentrations in both bread types. Moreover, five compounds were present in the HPF of both bread types, and of these, ferulic and sinapic acids showed higher concentrations in QB (around 8- and 13-fold increments, respectively), caffeic, and *p*-coumaric were equivalent in both breads, and naringenin resulted more abundant in WB. QB also stood out for the occurrence of specific compounds that were not detected in WB, particularly *p*-coumaric, ferulic, epicatechin, hyperoside, quercetin-3-*O*-glucopyranoside, and rutin in the EPF, and 2,4-dihydroxybenzoic, rosmarinic, syringic, and epigallocatechin in the HPF.

The group of PAs exhibited by far the highest content in QB. Following the trend found in QF, *p*-hydroxybenzoic was the most abundant PA in the EPF (constituted 87%). Relevant concentrations were found in this fraction by *p*-coumaric, vanillic, and ferulic acids. The compounds identified in the EPF also appeared in the HPF, except for *p*-hydroxybenzoic acid, gallic acid, vanillic acid, and chlorogenic acid. Ferulic acid stood out in the HPF for being around 74% of the polyphenol content in this fraction, followed far behind by sinapic (15.8%) and syringic acids (5.7%). FLs were almost nonexistent in the HPF and mainly presented low concentrations in the EPF, except for quercetin and rutin that respectively constituted 63% and 30% of FLs_EPF_ contents.

The more abundant compounds in QB, namely *p*-hydroxybenzoic acid, quercetin, and rutin (in the EPF) and acids ferulic and sinapic (in the HPF), are dietary polyphenols with potential biological activities. Several appreciable therapeutic roles have been attributed to them for their antioxidant capacity, and they could, hence, confer bread interesting functional properties [39,40,41,42,43,44]. Some in vivo studies have examined their potential biological effects. For instance, Kim et al. showed that as *p*-hydroxybenzoic acid enhances antioxidant enzyme activities, as intracellular ROS levels lower, the life span in the nematode *Caenorhabditis elegans* model system prolongs [45]. No studies addressing the effect of this compound on animal or preclinical disease models have been found in literature searches. Many studies in animals and humans that refer to quercetin and rutin show their anticancer, anti-inflammatory, antidiabetic, and anti-degenerative activities, among others (see the reviews on the subject and references therein: [39,43,46,47,48,49]). Recently, Lai and Wong [50] reviewed the interest in the optimization of quercetin-based functional foods and referred to clinical studies that support the health benefits of the oral intake of this flavonoid. Ferulic acid in QB appeared mainly in a bound form (95% of the total amount of ferulic) following the same behavior as in flours (Table 2) and is consistent with what is reported for this compound in cereals [48]. As generally described for bound forms based on their low absorption, it is assumed that a large proportion of bound ferulic forms directly reach the large intestine, where they can exert antioxidant action and even modulate the bacterial population. This can be especially relevant in colorectal malignancies [51]. Alazzouni et al. have shown the therapeutic activity of ferulic acid in rats with colon cancer [52]. Microflora modulating action in the colon has also been described for sinapic acid [53]. This phenolic acid was the second predominant compound in the HPF so the developed bread could also have this effect. Apart from the local action of bound forms, it has been suggested that part of bound polyphenol compounds is digested and can be absorbed in small and large intestines to some extent with putative beneficial effects on extraintestinal diseases [54]. In fact, diabetes mellitus protection and a neuroprotective role, among other effects, have been attributed to ferulic acid in animal models [55,56], and Russo et al. found that high ferulic acid intake significantly reduces the risk of prostate cancer in a human study [57]. The potential beneficial effects of sinapic acid at the extraintestinal level have also been investigated and reported in preclinical studies (see the work of [44] and references therein). In view of all this information, QB could be useful for reducing the risk of oxidative stress-related diseases, which would be alleviated by predominant individual polyphenol compounds and even by compounds present at moderate concentrations. As stressed in the introduction, polyphenols’ capacity to promote positive health effects is dependent on their chemical structure, bioavailability, absorption, and gut microbiota interaction [21,22]. A small polyphenols percentage is absorbed in the upper digestive tract, but most of them reach the colon, where they are transformed by the microbiota into absorbable compounds [21,22]. This is the case of quercetin, which health-promoting properties it is known that they are due to microbiota-mediated metabolites [58]. Moreover, polyphenols in the colon can concomitantly affect the microbial composition. Examples of positive and negative effects on bacteria composition have been recently reported for many pure polyphenols ([22] and references therein). For instance, quercetin has the ability to inhibit some pathogenic bacteria such as *Escherichia coli* or *Staphylococcus* species, and rutin enhances the growth of beneficial species as *Lactobacillus* and *Bifidobacterium*. Due to all these considerations and other factors such as polyphenols synergism, food matrix interaction, and food processing, oral administration of individual compounds generally differs from the intake of dietary phenolic compounds [59]. There is no preclinical or clinical evidence for the beneficial effects of quinoa-containing breads consumption in relation to its antioxidant properties. However, several research works have focused on quinoa seeds and quinoa-derived products other than bread. They have reported hypolipidemic and antidiabetic potentials and changes in antioxidant-related biomarkers (reduced oxidative stress and increased antioxidant defenses) (the work of [9] and references therein). Taken together, the data obtained and literature evidence suggest that quinoa-containing breads could positively contribute to human health upon consumption.

### 3.3. Impact of the Bread-Making Process on Phenolic Content and Individual Compounds

**Figure 1:**
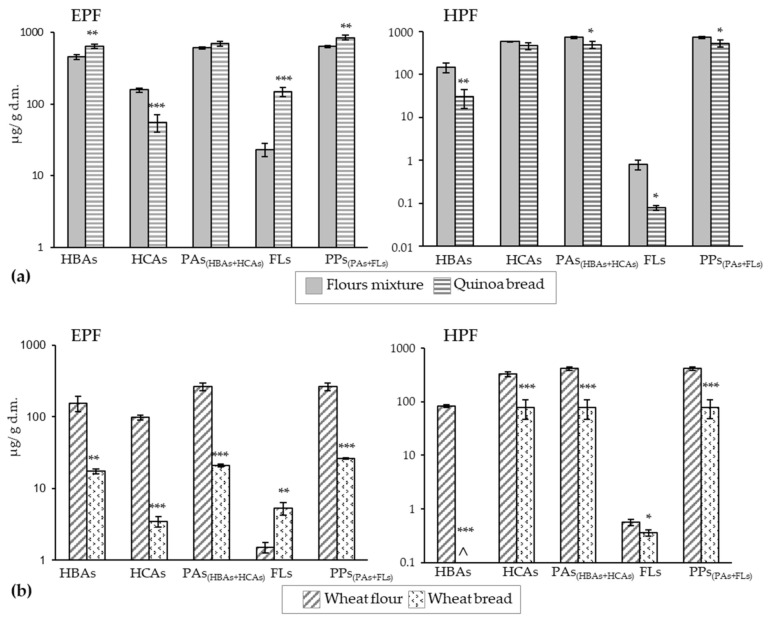
Effect of the bread-making process on the concentration of the different groups of polyphenols at the extractable (EFP) and hydrolyzable (HPF) fractions of 25% quinoa bread (**a**) and wheat bread (control) (**b**). Comparisons were always made between the flours mixture (75% wheat + 25% quinoa) and the quinoa bread values (**a**) and between the wheat flour and wheat bread values (**b**) in each group of compounds. Values are the sum of the corresponding individual compounds’ concentrations (Table 2 and Table 3). *, **, *** denote significances at *p* < 0.05, *p* < 0.01 and *p* < 0.001, respectively. HBA: hydroxybenzoic acids, HCA: hydroxycinnamic acids, PAs: phenolic acids, FLs: flavonoids, PPs: polyphenols, ^ not detected.

Figure 1a shows the bread-making effect on the content of major polyphenol groups (HBAs, HCAs, FLs) and of PPs, calculated as the sum of the concentrations of individual compounds in both the EPF and HPF of QB. Comparisons were made between the concentration values calculated for the flours mixture according to those previously determined in QFs and WFs (Table 2 and Table 3) and the concentrations of the same compounds determined in bread. Statistically significant changes in the concentration of some polyphenolic groups between the flours mixture and QB were detected.

The main effect was an increase in the FLs content in the EPF (6.4-fold higher in QB than in the flours mixture). Significant increases were also observed in the EPF of QB for HBAs (1.4-fold) and PPs (1.3-fold). Conversely, the corresponding contents in the HPF lowered (by 5-, 10.6-, and 1.5-fold in QB than in the flours mixture for HBAs, FLs, and PPs, respectively). Several authors describe increments in the EPF levels in quinoa seeds [60] and other grains [48] after fermentation and thermal processing, and Abdel-Aal and Rabalski reported larger amounts of free PAs and smaller amounts of bound ones in baked products [61]. All these studies attribute this effect on free and bound forms levels to the release of the phenolic compounds originally bound to cell walls by becoming part of the soluble-extractable fraction. HCAs were the least stable polyphenols in the EPF, whose concentration significantly dropped in bread (2.9-fold less in QB than in the flours mixture). However, due to the significant increase in HBAs, the content of free PAs (HBAs + HCAs) did not significantly alter after bread making. Carciochi et al. proposed greater thermal stability of free HBAs compared to HCAs based on chemical structure [60]. Food matrix characteristics also play an important role in the stability of phenolic compounds; for instance, fat seems to make compounds accessibility difficult and, therefore, product degradation by heating lessens [61]. Ballester-Sánchez et al. reported that black QB showed a higher lipid content than WB (around twice more) [12]. This could partly explain the different behavior displayed by WB compared to QB under the same bread-making conditions (Figure 1b). Considerable drops in the EPF of WB occurred (HBAs: 9-fold, HCAs: 29-fold, PAs: 12.5-fold, PPs: 10-fold) that far exceeded those of QB, and only the free FLs forms were enhanced. This could indicate the greater thermolability of the soluble-free compounds in this matrix. Losses were also more pronounced in the HPF fraction of WB than in that of QB, and HBAs were undetectable after bread making.

**Figure 2:**
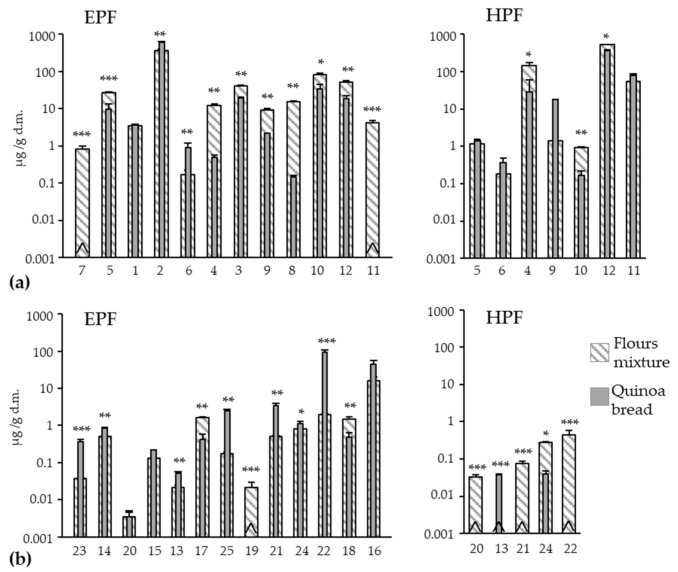
Effect of the bread-making process on the content of each individual phenolic acid (**a**) and flavonoid (**b**) in the respective extractable (EPF) and hydrolyzable (HPF) fractions on quinoa bread. Comparisons were always made between the flours mixture (75% wheat + 25% quinoa) and the quinoa bread values in each compound. *, **, *** denotes significances at *p* < 0.05, *p* < 0.01 and *p* < 0.001, respectively. ^ not detected. 1 (gallic acid), 2 (*p*-hydroxybenzoic acid), 3 (vanillic acid), 4 (syringic acid), 5 (2,4-dihydroxybenzoic acid), 6 (rosmarinic acid), 7 (benzoic acid), 8 (chlorogenic acid), 9 (caffeic acid), 10 (*p*-coumaric acid), 11 (sinapic acid), 12 (ferulic acid), 13 (epigallocatechin), 14 (catechin), 15 (epicatechin), 16 (rutin), 17 (hyperoside), 18 (quercetin-3-O-glucopyranoside), 19 (kaempferol-3-O-D-glucoside), 20 (daidzein), 21 (luteolin), 22 (quercetin), 23 (apigenin), 24 (naringenin), 25 (kaempferol).

The bread-making effect on the concentration of individual compounds brought about a different behavior between PAs and FLs. The PAs group generally decreased in the EPF (Figure 2a); only 2 compounds were significantly enhanced (*p*-hydroxybenzoic and rosmarinic acids with increments of 64% and 435%, respectively), while 9 of the 12 compounds present in the flours mixture diminished. Syringic and chlorogenic acids underwent marked reductions (96% and 99%, respectively) followed by caffeic (77%) and benzoic and sinapic acids were undetectable in the QB. Four compounds obtained similar reduction percentages, between 53% and 65% (vanillic < *p*-coumaric < ferulic < 2,4-dihydroxybenzoic), while gallic remained unaltered. A drop in the concentrations of soluble forms can be explained by the different mechanisms that occur during the bread-making process. For example, oxidation phenomena during kneading and/or thermal sensitivity, which imply chemical transformation or degradation [60,61,62,63,64]. The formation of complexes with other compounds such as proteins can occur during bread making, which would decrease their extractability and, hence, their concentrations in bread [63]. The bound syringic, *p*-coumaric, and ferulic acids obtained statistically significant reduction rates (81%, 74%, and 30%, respectively), but this behavior did not correspond to the increments in their respective free forms, which could be expected. This could be because the conversion from these bound forms into the corresponding soluble-extractable form was not intense enough to counteract the degradation of the latter by any of the aforementioned mechanisms.

For the individual FLs in the EPF (Figure 2b), and unlike that observed for PAS, concentrations considerably rose after the bread-making process (7 of the 13 compounds were enhanced).

The increments in apigenin (876%), kaempferol (1304%), luteolin (580%), and quercetin (4762%) were especially notable. The kaempferol-3-*O*-D-glucoside completely disappeared in QB, and its degradation could cause the marked gain in kaempferol. The luteolin and quercetin increments could account partly for the hydrolysis of the corresponding bound forms as they disappeared or were undetectable in the HPF. Degradation of quercetin’s conjugate forms detected in the EPF (hyperoside, also called quercetin-3-*O*-galactoside, and quercetin-3-*O*-glucopyranoside) could have also contributed to a remarkable gain in free quercetin; these conjugates decreased by 74% and 68%, respectively. During bread making, rutin (synonym: quercetin rutinoside) has been reported to be partly transformed into quercetin [65,66]. This behavior was observed in the control bread (WB), rutin totally degraded, and quercetin gained around 43-fold (Table 3). Nevertheless, in QB, the rutin concentration did not lower. The matrix type probably influences the stability of some polyphenols by leading to changes in their degradation [61]. The occurrence of some polyphenols in larger proportions in bread than in flours could be due to enhanced stability as a result of their interaction with polysaccharides of flours [67]. In addition to those aforementioned FLs, others with a significantly enhanced concentration in the EPF of the QB were catechin (65%), epigallocatechin (146%), and naringin (41%), while daidzein and epicatechin remained statistically unaltered.

### 3.4. Antioxidant Activity

**Figure 3:**
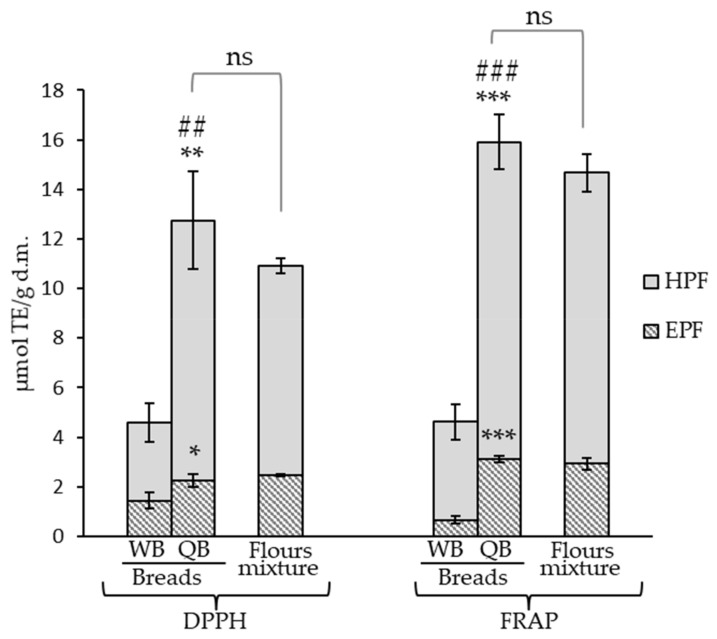
Antioxidant activity of the control bread (WB) and quinoa bread (QB) and effect of the bread-making process on the antioxidant activity of QB (flours mixture vs. QB). *, **, *** denote significances at *p* < 0.05, *p* < 0.01, and *p* < 0.001, respectively, when the extractable (EPF) and hydrolyzable polyphenols fractions (HPF) from QB were compared to the respective control bread (WB) fractions. ^##^ *p* < 0.01, ^###^ *p* < 0.001 when EPF + HPF sum was compared. ns: no statistically significant differences when the antioxidant activity of the flours mixture (75% wheat + 25% black quinoa) was compared to QB at the EPF, HPF, and EPF + HPF levels.

Antioxidant capacity in WB and QB and in their respective flours was evaluated by the DPPH and FRAP in vitro methodologies. The antioxidant capacity of QB was significantly greater than that of the control WB in both EPF and HPF and by both the tested in vitro methodologies (Figure 3). The results obtained by DPPH and FRAP were similar, although values in QB’s EPF and HPF were slightly higher by FRAP (e.g., 1.24-fold higher when the sum of antioxidant activities due to both fractions, EPF + HPF, was considered) and the same behavior was observed when breads were compared. Therefore, although the compounds present in the quinoa-containing bread had the ability to quench DPPH radicals and to reduce potential based upon the ferric ion, the data suggested that this last mechanism was somewhat more important.

The results confirmed that the incorporation of 25% quinoa into bread is sufficient to achieve a significant increase in bread’s antioxidant capacity and agree with others studies that describe the presence of phenolic compounds generally contributes significantly to the antioxidant potential [68]. Greater antioxidant activity in quinoa-containing breads compared with the control has been described in the EPF from wheat bread with 15% and 30% quinoa [16]. These authors described DPPH and FRAP values in the QB (DPPH: 1.17 µm/g and FRAP: 73.75 mg Trolox/100 g, i.e., 2.095 µmol/g) of the same order as our results. Concerning the HPF, the present work and our previous results [17] are the first studies taking into account the antioxidant activity of these fractions in QB.

The effect of the bread making was evaluated by comparing QB to flour mixtures (Figure 3). The process did not lead to significant changes in the antioxidant capacity of the EPF and HPF, although it has been reported that the active antioxidant compounds present in flour could be damaged or degraded by the baking process [5]. Furthermore, an upward trend was observed, the antioxidant capacity of the HPF and hence that of EPF + HPF resulted slightly enhanced, and despite not being statistically significant, this pattern was observed with the application of both in vitro methodologies. This could be due to the formation of Maillard reaction products during baking as hydroxymethylfurfural, previously described in this type of bread [17]. As these products are antioxidant agents [5], their formation could contribute to counteracting possible losses in the antioxidant activity.

## 4. Conclusions

The present research work confirmed our previous findings of the potential of black QF being used as an ingredient to improve the polyphenolic content and antioxidant activity of WB-based baked products [17]. The phenolic profile of WF and QF vastly differed and, together with the changes derived from the bread-making process, defined the resulting breads. The general improvement of the quinoa-containing bread compared to WB was evident; QB presented improved total polyphenols content, i.e., considering both the EPF and HPF compared to the control (12.8-fold more) and showed antioxidant capacity increases of up to 3-fold compared to the WB. Several studies have shown that the occurrence of high total phenolic composition and antioxidant activity are associated with more significant biological effects [69]. In view of the protective roles attributed to the polyphenol compounds predominantly found in QB, this bakery food can be considered a contributor to promoting positive health effects in daily diets. Moreover, the resulting bread offers additional benefits for consumers, as we have previously shown, by general improving bread’s nutritional profile [12]. From what we know, this is the first study to deal with the chromatographic polyphenolic profile of refined-wheat bread partially enriched with QF. The scarce studies reported to date have only taken into account the impact of adding QF on the polyphenol content and antioxidant effect [14,15,16,17], or they identify polyphenolic compounds, but in QB formulated without WF [14] or in bread enriched with quinoa leaves [70]. However, polyphenol content and composition determinations partially reflect the biological activity of the bread by not considering their bioavailability. Polyphenols’ effectiveness is strongly influenced by their intestinal absorption, metabolism, and subsequent activity in target tissues; thus, bioavailability is a pivotal factor to assess the potential health effect of food matrix containing bioactive compounds [20]. To evaluate the beneficial effects that QB consumption appears to possess, future in vitro and in vivo studies involving bioavailability are needed. The gut microbiota can metabolize polyphenolic compounds and influence their absorption [51], being another important factor to be considered in further investigations to improve the understanding of quinoa-containing bread potential functionality.

## Figures and Tables

**Table 1:** Phenolic compounds identified in the extractable (EPF) and hydrolyzable (HPF) fractions of raw materials (wheat flour: WF and quinoa flour: QF) and bread (wheat bread: WB and 25% quinoa bread: QB).

Peak No.	Name	Molecular Formula	Ms [M−H]^−^ (*m/z*)	R_t_ (min)	MS Fragments	Type of Extract (Sample)
Hydroxybenzoic acids						
1	Gallic acid	C_7_H_6_O_5_	169.01	2.06	123, 106	EPF (WF, QF, WB, QB)
2	*p*-HBA ^a^	C_7_H_6_O_3_	137.02	3.62	121	EPF (WF, QF, WB, QB)
3	Vanillic acid	C_8_H_8_O_4_	167.03	4.00	151, 122 107	EPF (WF, QF, WB, QB)
4	Syringic acid	C_9_H_10_O_5_	197.04	4.07	181, 166, 122	EPF (WF, QF, WB, QB); HPF (WF, QF, ---, QB)
5	2,4-DHBA ^a^	C_7_H_6_O_4_	153.01	4.13	137, 108	EPF (WF, QF, WB, QB); HPF (WF, QF, ---, QB)
6	Rosmarinic acid	C_18_H_16_O_8_	359.08	5.51	197, 179, 161	EPF (WF, QF, WB, QB); HPF (---, QF, ---, QB)
7	Benzoic acid	C_7_H_6_O_2_	121.03	5.83	77	EPF (WF, ---, ---, ---)
Hydroxycinnamic acids						
8	Chlorogenic acid	C_16_H_18_O_9_	353.09	3.34	191, 179	EPF (WF, ---, WB, QB)
9	Caffeic acid	C_9_H_8_O_4_	179.03	3.98	135, 118, 107	EPF (WF, QF, WB, QB); HPF (WF, ---, WB, QB)
10	*p*-Coumaric acid	C_9_H_8_O_3_	163.04	4.85	118, 96, 92	EPF (WF, QF, ---, QB); HPF (WF, QF, WB, QB)
11	Sinapic acid	C_11_H_12_O_5_	223.06	5.02	207, 193, 149	EPF (WF, QF, ---, ---); HPF (---, QF, WB, QB)
12	Ferulic acid	C_10_H_10_O_4_	193.05	5.08	178, 133, 116	EPF (WF, QF, ---, QB); HPF (WF, QF, WB, QB)
Flavonoids						
13	Epigallocatechin	C_15_H_14_O_7_	305.07	3.20	287, 179, 121	EPF (WF, QF, WB, QB); HPF (---, ---, ---, QB)
14	Catechin	C_15_H_14_O_6_	289.07	3.55	257, 203, 123	EPF (WF, QF, WB, QB)
15	Epicatechin	C_15_H_14_O_6_	289.07	3.97	242, 203, 179	EPF (WF, QF, ---, QB)
16	Rutin	C_27_H_30_O_16_	609.15	4.51	300, 271, 255	EPF (WF, QF, ---, QB)
17	Hyperoside	C_21_H_20_O_12_	463.08	4.71	301	EPF (---, QF, ---, QB)
18	Q3G ^a^	C_21_H_20_O_12_	463.08	4.75	301	EPF (---, QF, ---, QB)
19	K3G ^a^	C_21_H_20_O_11_	447.09	5.17	311, 285, 255	EPF (WF, ---, ---, ---)
20	Daidzein	C_15_H_10_O_4_	253.05	6.44	224, 192, 132	EPF (WF, QF, WB, QB); HPF (WF, ---, ---, ---)
21	Luteolin	C_15_H_10_O_6_	285.04	6.70	203, 175, 151	EPF (WF, QF, WB, QB); HPF (---, QF, ---, ---)
22	Quercetin	C_15_H_10_O_7_	301.03	6.77	255, 239	EPF (WF, QF, WB, QB); HPF (---, QF, ---, ---)
23	Apigenin	C_15_H_10_O_5_	269.05	7.50	209, 151, 117	EPF (WF, QF, WB, QB)
24	Naringenin	C_15_H_12_O_5_	271.06	7.52	177, 151, 119	EPF (WF, QF, WB, QB); HPF (WF, QF, WB, QB)
25	Kaempferol	C_15_H_10_O_6_	285.04	7.67	203, 185, 151	EPF (---, QF, WB, QB); HPF (---, ---, WB, ---)

^a^ Abbreviations: *p*-HBA: *p*-hydroxybenzoic 2,4-DHBA: 2,4-dihydroxybenzoic, Q3G: quercetin-3-O-glucopyranoside, K3G: kaempferol-3-O-D-glucoside.

**Table 2:** Phenolic acids quantification (µg/g) in raw materials (wheat and quinoa flours) and bread (control and 25% quinoa).

Compound	FLOURS				BREAD			
	EPF		HPF		EPF		HPF	
	Wheat	Quinoa	Wheat	Quinoa	Wheat	Quinoa	Wheat	Quinoa
HYDROXYBENZOIC ACIDS								
Benzoic	1.10 ± 0.21	n.d.	n.d.	n.d.	n.d.	n.d.	n.d.	n.d.
2,4-DHBA	31.8 ± 2.1	13.8 ± 1.9 **	0.71 ± 0.21	1.31 ± 0.15	2.06 ± 0.89	9.56 ± 3.6	n.d.	1.43 ± 0.05
Gallic	3.49 ± 0.65	3.18 ± 0.71	n.d.	n.d.	1.58 ± 0.05	3.62 ± 0.23 *	n.d.	n.d.
*p*-HBA	49.1 ± 7.8	1282 ± 56 ***	n.d.	n.d.	12.3 ± 2.0	611 ± 38 ***	n.d.	n.d.
Rosmarinic	0.04 ± 0.00	0.56 ± 0.20	n.d.	0.74 ± 0.51	0.18 ± 0.07	0.99 ± 0.25 *	n.d.	0.36 ± 0.27
Syringic	16.0 ± 1.8	0.09 ± 0.04 **	82.24 ± 5.6	168 ± 43 *	0.33 ± 0.04	0.50 ± 0.07	n.d.	28 ± 14
Vanillic	45.7 ± 2.2	25.4 ± 3.3 **	n.d.	n.d.	1.25 ± 0.25	19.1 ± 1.7 ***	n.d.	n.d.
Sum of HBAs ^a^	147.2 ± 8.5	1325 ± 56 ***	82.9 ±5.6	170 ± 43 *	17.7 ± 2.2	645 ± 38 ***		30 ± 14
HYDROXYCINNAMIC ACIDS								
Caffeic	11.8 ± 1.1	2.17 ± 0.20 ***	1.90 ± 0.01	n.d.	0.37 ± 0.10	2.17 ± 0.06 ***	24.2 ± 9.2	18.1 ± 7.2
Chlorogenic	20.9 ± 1.0	n.d.	n.d.	n.d.	3.01 ± 0.37	0.15 ± 0.01 **	n.d.	n.d.
*p*-Coumaric	2.1 ± 0.4	322 ± 36 ***	1.66 ± 0.31	0.70 ± 0.09 *	n.d.	35 ± 11	0.34 ± 0.13	0.25 ± 0.05
Ferulic	57.6 ± 5.6	35.5 ± 8.3 *	324 ± 34	587 ± 32 *	n.d.	18.8 ± 3.7	48 ± 17	367 ± 49 **
Sinapic	5.58 ± 0.95	0.09 ± 0.03 **	n.d.	223 ± 46	n.d.	n.d.	5.8 ± 2.2	78 ± 15 *
Sum of HCAs ^a^	98.0 ± 5.9	359 ± 37 **	328 ± 35	811 ± 56 ***	3.38 ± 0.38	56 ± 12 **	78 ± 21	463 ± 52 **
Sum of Pas ^a^	245 ± 10	1685 ± 67 ***	411 ± 35	982 ± 71 ***	21.1 ± 2.2	700 ± 40 ***	78 ± 20A	493 ± 54 **

Comparisons were always made between flour values (quinoa flour vs. wheat flour) and between bread values (quinoa bread vs. wheat bread) in each type of extract (EPF: extractable polyphenols fraction or HPF: hydrolyzable polyphenols fraction). Data are expressed as mean ± standard deviation. *, **, *** denotes significances at *p* < 0.05, *p* < 0.01, and *p* < 0.001, respectively. ^a^ Abbreviations: 2,4-DHBA: 2,4-dihydroxybenzoic; *p*-HBA: *p*-hydroxybenzoic; HBAs: hydroxybenzoic acids; HCAs: hydroxycinnamic acids; PAs: phenolic acids (HBAs + HCAs). n.d. not detected.

**Table 3:** Flavonoids quantification (µg/g) in raw materials (wheat and quinoa flours) and bread (control and 25% quinoa).

Compound	FLOURS				BREAD			
	EPF		HPF		EPF		HPF	
	Wheat	Quinoa	Wheat	Quinoa	Wheat	Quinoa	Wheat	Quinoa
Apigenin	0.019 ± 0.003	0.095 ± 0.001 ***	n.d.	n.d.	0.87 ± 0.12	0.367 ± 0.046 **	n.d.	n.d.
Catechin	0.594 ± 0.014	0.240 ± 0.045 ***	n.d.	n.d.	0.0344 ± 0.016	0.833 ± 0.050 ***	n.d.	n.d.
Daidzein	0.004 ± 0.001	0.001 ±0.000	0.04 ± 0.005	n.d.	0.005 ± 0.001	0.004 ± 0.002	n.d.	n.d.
Epicatechin	0.051 ± 0.032	0.38 ± 0.12	n.d.	n.d.	n.d.	0.220 ± 0.003	n.d.	n.d.
EGC ^a^	0.013 ± 0.009	0.045 ± 0.010	n.d.	n.d.	0.065 ± 0.017	0.052 ± 0.005	n.d.	0.038 ± 0.002
Hyperoside	n.d.	6.60 ± 0.34	n.d.	n.d.	n.d.	0.43 ± 0.15	n.d.	n.d.
Kaempferol	n.d.	0.704 ± 0.063	n.d.	n.d.	0.189 ± 0.032	2.471 ± 0.28 ***	0.267 ± 0.044	n.d.
K3G ^a^	0.134 ± 0.021	n.d.	n.d.	n.d.	n.d.	n.d.	n.d.	n.d.
Luteolin	0.093 ± 0.001	1.757 ± 0.089 ***	n.d.	0.310 ± 0.040	2.60 ± 0.52	3.46 ± 0.56	n.d.	n.d.
Naringenin	0.053 ± 0.007	3.095 ± 0.036 ***	0.264 ± 0.027	0.275 ± 0.024	0.065 ± 0.008	1.15 ± 0.12 ***	0.094 ± 0.000	0.040 ± 0.009 *
Quercetin	0.034 ± 0.007	7.69 ± 0.54 ***	n.d.	1.42 ± 0.17	1.48 ± 0.31	95 ± 13 ***	n.d.	n.d.
Q3G ^a^	n.d.	6.01 ± 0.69	n.d.	n.d.	n.d.	0.48 ±0.17	n.d.	n.d.
Rutin	0.67 ± 0.21	62 ± 16 **	n.d.	n.d.	n.d.	45 ± 14	n.d.	n.d.
Sum of FLs ^a^	1.55 ± 0.21	89 ± 16 **	0.31 ± 0.03	1.99 ± 0.18 **	5.30 ± 0.62	149 ± 18 **	0.360 ± 0.040	0.080 ± 0.010 ***
Sum of PPs ^a^	247 ± 10	1774 ± 69 ***	411 ± 35	984 ± 71 ***	26.4 ± 2.3	850 ± 44 ***	79 ± 21	493 ± 54 **

Comparisons were always made between flour values (quinoa flour vs. wheat flour) and between bread values (quinoa bread vs. wheat bread) in each type of extract (EPF: extractable polyphenols fraction or HPF: hydrolyzable polyphenols fraction). Data are expressed as mean ± standard deviation. *, **, *** denotes significances at *p* < 0.05, *p* < 0.01, and *p* < 0.001, respectively. ^a^ Abbreviations: EGC: epigallocatechin, K3G: kaempferol-3-*O*-D-glucoside, Q3G: quercetin-3-*O*-glucopyranoside, FLs: flavonoids, PPs: polyphenols (PAs Table 2 + FLs).

## Data Availability

Data is contained within the article.
